# RETRACTION: Evaluating the Efficacy of Combined Flap Coverage, Antibiotic‐Loaded Bone Cement and Negative Pressure Irrigation in Traumatic Osteomyelitis Management

**DOI:** 10.1111/iwj.70627

**Published:** 2025-04-21

## Abstract

**RETRACTION:** CongB. and ChenM. , “Evaluating the Efficacy of Combined Flap Coverage, Antibiotic‐Loaded Bone Cement and Negative Pressure Irrigation in Traumatic Osteomyelitis Management,” International Wound Journal 21, no. 1 (2024): e14650, 10.1111/iwj.14650.38272791 PMC10794078

The above article, published online on 17 January 2024, in Wiley Online Library (http://onlinelibrary.wiley.com/), has been retracted by agreement between the journal Editor in Chief, Professor Keith Harding; and John Wiley & Sons Ltd. Following an investigation by the publisher, all parties have concluded that this article was accepted solely on the basis of a compromised peer review process. The editors have therefore decided to retract the article. The authors did not respond to our notice regarding the retraction.

**RETRACTION:** B. Cong and M. Chen , “Evaluating the Efficacy of Combined Flap Coverage, Antibiotic‐Loaded Bone Cement and Negative Pressure Irrigation in Traumatic Osteomyelitis Management,” International Wound Journal 21, no. 1 (2024): e14650, 10.1111/iwj.14650.38272791 PMC10794078

The above article, published online on 17 January 2024, in Wiley Online Library (http://onlinelibrary.wiley.com/), has been retracted by agreement between the journal Editor in Chief, Professor Keith Harding; and John Wiley & Sons Ltd. Following an investigation by the publisher, all parties have concluded that this article was accepted solely on the basis of a compromised peer review process. The editors have therefore decided to retract the article. The authors did not respond to our notice regarding the retraction.

